# The Interplay of HIV-1 and Macrophages in Viral Persistence

**DOI:** 10.3389/fmicb.2021.646447

**Published:** 2021-04-07

**Authors:** Chynna M. Hendricks, Thaissa Cordeiro, Ana Paula Gomes, Mario Stevenson

**Affiliations:** ^1^Department of Microbiology & Immunology, Miller School of Medicine, University of Miami, Miami, FL, United States; ^2^Department of Medicine, Miller School of Medicine, University of Miami, Miami, FL, United States

**Keywords:** HIV, reservoirs, macrophages, latency, functional cure, host factors

## Abstract

HIV-1 has evolved mechanisms to evade host cell immune responses and persist for lifelong infection. Latent cellular reservoirs are responsible for this persistence of HIV-1 despite the powerful effects of highly active antiretroviral therapies (HAART) to control circulating viral load. While cellular reservoirs have been extensively studied, much of these studies have focused on peripheral blood and resting memory CD4+ T cells containing latent HIV-1 provirus; however, efforts to eradicate cellular reservoirs have been stunted by reservoirs found in tissues compartments that are not easily accessible. These tissues contain resting memory CD4+ T cells and tissue resident macrophages, another latent cellular reservoir to HIV-1. Tissue resident macrophages have been associated with HIV-1 infection since the 1980s, and evidence has continued to grow regarding their role in HIV-1 persistence. Specific biological characteristics play a vital role as to why macrophages are latent cellular reservoirs for HIV-1, and *in vitro* and *in vivo* studies exhibit how macrophages contribute to viral persistence in individuals and animals on antiretroviral therapies. In this review, we characterize the role and evolutionary advantages of macrophage reservoirs to HIV-1 and their contribution to HIV-1 persistence. In acknowledging the interplay of HIV-1 and macrophages in the host, we identify reasons why current strategies are incapable of eliminating HIV-1 reservoirs and why efforts must focus on eradicating reservoirs to find a future functional cure.

## Introduction

The HIV-1 pandemic persists despite the revolution of suppressive antiretroviral therapy (ART) in controlling HIV-1 replication. Recrudescing viremia emerges from cellular reservoirs once suppressive ART has ceased, as well as from compartments unaffected by ART. Latent cellular reservoirs are the major obstacle in preventing HIV-1 eradication and further characterization and understanding is necessary for cure efforts. Resting memory CD4+ T cells reservoirs are the main focus of HIV-1 reservoir studies, overlooking other CD4-containing cell types, such as myeloid cells. Myeloid cells, specifically macrophages, have been associated with HIV-1 infection since the 1980s (Ho et al., 1986; Koenig et al., 1986). Residual viremia from cART-suppressed patients validates that small populations of viruses are not genetically identical to the proviruses found in resting CD4+ T cells but are from an unknown source (Bailey and Hutter, 2006; Brennan et al., 2009; Vibholm et al., 2019). Furthermore, a minor population of viruses from macrophage reservoirs appears in rebounding viremia from reservoirs that predate the initiation of cART, which suggests macrophages are infected early on and assist in the persistence of HIV-1 infection (Andrade et al., 2020). Since the 1990s, evidence has implicated macrophages assist in HIV-1 persistence *in vivo* and produce virions *in vitro* (Perelson et al., 1997).

Macrophages, like CD4+ T cells, express CD4 and chemokine coreceptors CCR5 and CXCR4 on their cell surface allowing for HIV-1 susceptibility. Macrophages are susceptible to CCR5-tropic and dual-tropic viral infection, but determinants that assist in tropism are much more complicated than coreceptor usage (Borrajo et al., 2019). Specifically, macrophages express significantly lower levels of CD4, meaning macrophage-tropic viruses must have a high degree of affinity to CD4 to mediate fusion with the macrophage cell membrane (Kazazi et al., 1989; Joseph et al., 2014). Macrophages are permissive to productive infection both *in vivo* and *in vitro*. Furthermore, genetically related Simian Immunodeficiency Virus (SIV) has been shown to infect simian macrophages (Ho et al., 1986; Koenig et al., 1986; Li et al., 2015). These HIV-1 and SIV infected macrophages have been found in various tissue compartments *in vivo*, including the central nervous system (CNS), lymph nodes, gut lymphoid-associated tissue (GALT), and lungs (Albright et al., 2000, 2003; Igarashi et al., 2001; Jambo et al., 2014; Li et al., 2015; Avalos et al., 2016). While HIV-1 infects and sustains infection in macrophages, the contribution of macrophages as a viable reservoir remains in question.

Investigating the role of macrophages in HIV-1 persistence is challenging. Macrophages are heterogenous, non-dividing cells that are influenced by their surroundings, which can make infected macrophages unique to their niche (Gordon and Pluddemann, 2017). Tissue compartments in humans are not readily accessible unless donated from elective and necessary surgeries or post-mortem tissue. Some of these tissue compartments may provide immune sanctuaries, such as lymphoid tissues and the CNS to macrophage reservoirs (Kepler and Perelson, 1998; Smit et al., 2004; Fletcher et al., 2014). Experimental models, such as macrophage quantitative viral outgrowth assays (qVOA) and monocyte-derived macrophages (MDMs) models, have been developed to study macrophage reservoirs *in vitro*, while non-human primate and mice models are used to study macrophages *in vivo*. Yet, none of these models have grasped the complexity of macrophage heterogeneity in their contribution to HIV-1 persistence (Avalos et al., 2016; Honeycutt et al., 2016; Veazey and Lackner, 2017; Abreu et al., 2019).

While growing evidence supports the role of macrophage reservoirs, how they contribute to HIV-1 persistence is still largely unknown and characteristics of the reservoirs are still being understood. This gap in knowledge raises questions as to what extent macrophage reservoirs contribute to infection and if macrophage-tropic viruses truly originate from a macrophage reservoir. In this review, we address the biological characteristics and mechanisms of macrophage reservoirs in controlling HIV-1 latency and the factors that contribute to long term HIV-1 infection in relation to finding a functional cure.

## Macrophage Biology, HIV-1 Infection and Cellular Reservoirs

### Macrophage Heterogeneity Affects HIV-1 Infection

Macrophages are capable of sustaining HIV-1 infection due to a number of biological reasons. Firstly, macrophages are extraordinary plastic cells that exhibit extreme heterogeneity due to sensitivity to the local cytokine microenvironment (Mantovani et al., 2004; Sica and Mantovani, 2012; Gordon and Pluddemann, 2017). Macrophages are primed to install a particular, reversible phenotype and functional response based on cytokine stimuli and signals encountered in their microenvironment (Sica and Mantovani, 2012). This diversity allows macrophages to take on a wide variety of roles in innate immune response, phagocytosis, and tissue repair. Primary macrophages are challenging to investigate *in vivo*, thus *in vitro* models using MDMs have been created (Zalar et al., 2010; Yukl et al., 2013; Cribbs et al., 2015). Monocytes are isolated from peripheral blood and differentiated into MDMs that can be further stimulated in culture to obtain phenotypically distinct macrophages (Kruize and Kootstra, 2019). *In vitro* polarized macrophages can be classified as classically activated or inflammatory macrophages (M1) and alternatively activated or anti-inflammatory (M2) macrophages (Cassetta et al., 2011; Biswas et al., 2012; Mantovani et al., 2013; Schwartz et al., 2014; Sica et al., 2015; Murray, 2017).

**FIGURE 1:**
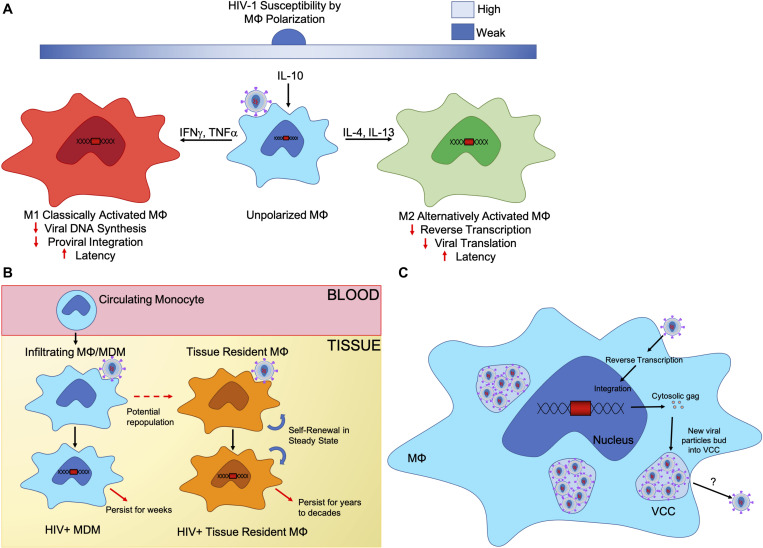
Biological characteristics of macrophage reservoirs. **(A)** Unpolarized macrophages are stimulated by the local cytokine milieu in different tissue compartments, which causes different macrophage polarization states. Macrophage polarization is a spectrum and reversible. Two extremes are the M1, classically activated or pro-inflammatory, macrophage and the M2, alternatively activated or anti-inflammatory, macrophage, who play different roles within the body. Upon HIV-1 infection, polarization states affect susceptibility of macrophages as well as inhibit viral replication at different points in the HIV-1 replication cycle. **(B)** Tissue resident macrophages are sustained by self-renewal, and in some tissue, infiltrating macrophages will replenish tissue resident macrophage populations. Both monocyte derived macrophages and tissue resident macrophages are susceptible to HIV-1 infection and can sustain long-term infection with tissue resident macrophages maintaining viral infection for years to decades. **(C)** Virus containing compartments (VCCs) are formed from the introduction of gag synthesis in the cytosol, promoting the formation of compartments. Viral particles assemble then bud into these VCCs, accumulating over time. Upon cell necrosis or stimulation from the microenvironment, VCCs may release their viral particles.

These distinct polarization states affect macrophage susceptivity to HIV-1 infection (Figure 1A). Macrophage polarization can range in acute and chronic stages of HIV-1 infection (Burdo et al., 2015). During acute infection, macrophages polarize to an M1 inflammatory state that eventually shifts to an M2 anti-inflammatory or immunosuppressive state in chronic infection (Becker, 2004; Li et al., 2009). Unpolarized macrophages are permissive to HIV-1 infection as they are not primed for a certain response. Different cytokine expressions during polarization leads both M1 and M2 macrophages to being refractory to HIV-1 infection, as well as impair HIV-1 viral functions during acute and chronic HIV-1 infection (Cassol et al., 2009, 2010; Galvao-Lima et al., 2017). During *in vitro* studies, IFNγ and TNFα stimulated M1 MDMs inhibited HIV-1 viral DNA synthesis, proviral integration, and transcription in comparison to unpolarized MDMs through an upregulation of APOBEC 3A in M1 MDMs (Cassetta et al., 2013). Furthermore, IFNγ polarized macrophages from the decidua basalis tissue in pregnant woman have been shown to be weakly permissive HIV-1 infection through cyclin-dependent kinase inhibitor p21Cip1/Waf1 and by toll-like receptor (TLR) 7 and TLR8 restriction of HIV-1 replication by further inducing IFNγ to maintain an M1 macrophage phenotype (El Costa et al., 2016). Primary MDMs stimulated IFNγ and TNFα into an M1 phenotype show viral containment and inhibition of viral replication and integration upon re-stimulation to an M1 phenotype. These M1-double stimulated MDMs have an upregulation in APOBEC3A and APOBEC3G restriction factors as well as negative regulators of proviral transcription, which assisted in keeping the replication-competent virus in a latent state (Graziano et al., 2018).

For M2 polarization, IL-4 and IL-13 polarized M2a macrophages can cause resistance to HIV-1 infection by limiting HIV-1 replication during reverse transcription of HIV-1 RNA (Schuitemaker et al., 1992; Montaner et al., 1997; Wang et al., 1998; Cobos Jimenez et al., 2012). IL-10 polarized M2c and TNFα polarized M1 macrophages control HIV-1 latency at the levels of viral replication at transcription and translation (Kootstra et al., 1994; Montaner and Gordon, 1994; Chang et al., 1996; Perez-Bercoff et al., 2003; David et al., 2006; Cassol et al., 2009; Cobos Jimenez et al., 2012). Although, macrophages in an intermediate polarization state (Mi)—somewhere between M1 and M2—were preferentially infected by HIV-1 in urethral tissue (Ganor et al., 2019). Additionally, macrophage susceptibility has been shown to differ amongst tissues due to mechanisms that are still debated. Heterogeneity of macrophages allows these cells to maintain different roles necessary for the tissue they reside, and the degree to which macrophages differ can be quite significant depending on their niche. Macrophages in the GALT are resistant to infection, whereas macrophages in the rectum and alveolar are readily permissive (King et al., 2013; McElrath et al., 2013; Jambo et al., 2014). Macrophage polarization helps to understand the complexities of macrophage heterogeneity *in vitro*; however, the plasticity of macrophages in different environments contributes to challenges around understanding how heterogeneity affects HIV-1 infection *in vivo*. Macrophage localization has proven to show drastically different susceptibilities to HIV-1 infection and mechanisms that may control latency, which suggests certain macrophage phenotypes provide better reservoirs.

### Macrophage Self-Renewal Potential and Latency

Tissue macrophages have the ability to sustain themselves. These macrophages have long been hypothesized as derived from monocytes; however, macrophages can be classified into two broad groups: tissue resident macrophages and infiltrating macrophages. The tissue resident macrophages perform homeostatic functions in their respective tissues in a steady state, while infiltrating macrophages are derived from circulating monocytes that infiltrate tissue and differentiate (Figure 1B) (Murray and Wynn, 2011). Early studies show bone marrow derived monocytes replenish tissue resident macrophages and that monocytes contribute to the population of lung alveolar macrophage replacement after they are depleted (van Furth and Cohn, 1968; Virolainen, 1968; Landsman et al., 2007). This has conflicted with evidence that primitive macrophages originate during embryonic stages developing before monocytes and are maintained as long-lived cells in tissue compartments (Takahashi et al., 1989). Microglia in the CNS are derived from these embryonic macrophages and are maintained independently from monocytes through self-renewal in a steady state (Schulz et al., 2012).

Tissue resident macrophages are seeded throughout tissues before birth during the embryonic state (Schulz et al., 2012; Hashimoto et al., 2013; Yona et al., 2013; Mass et al., 2016). Hashimoto et al. (2013) found that monocytes contribute very small portion to the tissue macrophage population to tissue macrophages in a steady state after cell turnover. In the lungs, bone marrow, and CNS, monocytes were found not to be the progenitors for tissue resident macrophages when in a steady state. Even when lung and splenic tissues were depleted of macrophages, they were repopulated by tissue resident macrophages independently of circulating monocytes, which suggests these macrophages are self-maintaining in the steady state and after cell turnover (Hashimoto et al., 2013). Soucie confirmed tissue resident macrophages are able to self-proliferate with minimal contribution from monocytes through a network of genes that control self-renewal potential in these mature cells (Soucie et al., 2016).

Tissue resident macrophages are long-lived innate immune cells that persist from weeks to decades. Infiltrating macrophages with no embryonic origins have estimated half-lives between 4 and 6 weeks, while tissue resident macrophages have a much slower turnover (Scott et al., 2014). Alveolar macrophages have a half-life around 2 months, while microglial cells can last between 4 years to decades (Cassol et al., 2006; Reu et al., 2017). These long-lived macrophages are suitable for maintaining HIV-1 infection in durations comparable to those of resting memory CD4+ T cells. For infected tissue resident macrophages, their ability to self-renew provides a mechanism for sustained HIV-1 infection in a long-lived population of cells throughout different compartments in the host. This provides the scaffolding to protect HIV-1 long term against the effects of ART as the virus goes quiescent and is self-maintained through the tissue macrophage population.

Additionally, acutely infected resting CD4+ T cells have an average half-life of 2 days, whereas infected monocytes and macrophages live significantly longer than their counterparts (Zhou et al., 2005; Koppensteiner et al., 2012). This poses the case that tissue macrophages may be able to maintain infection for a longer duration than CD4+ T cells. Furthermore, HIV-1 infected macrophages have evolved mechanisms to prevent cell death and prolong cell lifespans, thereby allowing macrophages to provide immune sanctuary to HIV-1 in the latent stage (Swingler et al., 2007; Reynoso et al., 2012). Due to the heterogenous nature of macrophages, specifics as to which population of macrophages is preferentially infected still remains unknown. In addition, the long lifespan and self-renewal capacity of macrophages means HIV-1 infection is sustained for lengthy, undisturbed periods of time.

### Macrophage Virus-Containing Compartments

As new virus is produced in infected cells, they require fusion, maturation, and assembly. The assembled virus retains the specific cellular membrane of the host cell, which derives the viral envelope. In infected lymphocytes, and certain cell lines like 293T and HeLa cells, HIV-1 buds straight through the cell plasma membrane (Jouvenet et al., 2006; Finzi et al., 2007). Alternatively, in HIV-1 infected macrophages, budding structures accumulate in subcellular compartments similar to endosomes (Orenstein et al., 1988; Raposo et al., 2002; Finzi et al., 2007). In these infected macrophages, pleomorphic vesicular structures containing viral-like particles have been identified (Ganor et al., 2019). When infected, MDMs produce an accumulation of viral particles in these vesicular structures (Deneka et al., 2007; Jouve et al., 2007). Different from CD4+ T cells, infected macrophages accumulate large internal vacuole containing virus, known as Virus-Containing Compartments (VCCs), that retain their infectious potential for extended periods of time and act as safe storage for infectious particles in a viral reservoir (Gaudin et al., 2013). Gaudin hypothesized that VCCs are formed upon HIV-1 infection. Intracellular gag is synthesized and accumulated in the cytosol to promote the formation of compartments made from the cell membrane. Viral particles are assembled in the cytosol and bud into these compartments, which eventually fill up the lumen (Figure 1C) (Gaudin et al., 2013). While HIV-1 gag is responsible for the formation of VCCs in macrophages, Hammond and peers found that cell surface lectin Siglec-1 is capable of attaching to the lipid envelopes of viruses, capturing the viral particles and forming VCCs, and these compartments allowed for direct transfer of virions to other target cells. Depletion of Siglec-1 decreases the production and size of VCCs in macrophages, suggesting the need for Siglec-1 in VCC formation (Hammonds et al., 2017).

The biological production of these structures allows for direct transfer of virus from infected cells in contact with uninfected cells. Macrophages favor retention of accumulated viral particles contained in VCC, triggering release through stimulation or cell necrosis or apoptosis. Upon stimulation by microenvironmental factors like extracellular ATP, macrophages are triggered to rapidly release infectious virions from VCCs (Graziano et al., 2018). Additionally, bone marrow stromal cell antigen 2 (BST2) has been found contained in VCCs with HIV-1 virions, tethering HIV-1 virions to the cell membrane or these VCCs. HIV-1 Vpu protein downregulates BST2 and removes it from VCCs, allowing for the expansion of VCCs with more viral particles (Leymarie et al., 2019).

The functions of VCCs remains debated. Gaudin noted an increase of density of intracellular viral gag in these compartments post-infection by immuno-EM (Gaudin et al., 2013). Additionally, these experiments highlighted a decrease in secretion of viral particles with their infectivity and transmission rate to CD4+ T cells decreasing overtime, thereby suggesting HIV-1 infected macrophages retain new virions in their compartment lumens (Gaudin et al., 2013). In a study of penile urethral tissue, Ganor et al. (2019) addressed the importance of VCCs in the presence of HIV-1 proteins, in p24, CD68 and CD4 stained urethral tissues on HIV-1/cART individuals. Stained tissue revealed p24 co-localized with macrophages co-expressing CD4 and CD68 in the urethral stroma. In contrast, urethral CD4+ T cells had no co-localization of p24 with high level CD4 expression. Evidence of VCC structures in urethral tissues supports macrophage reservoirs as sustaining HIV-1 in these tissues specifically (Ganor et al., 2019). These results demonstrate macrophages contain and shelter intact HIV-1 virions in VCC-like structures, which may act as an important viral reservoir specifically when viral capsid and virions could not be identified in CD4+ T cells. The inclusion of VCCs in macrophages allows for macrophages to maintain latent infection and to sustain active infection by release sheltered particles safe from ART. These sheltered particles make it possible for direct cell-to-cell infection of uninfected macrophages and CD4+ T cells in the same region as infected macrophages, which provides an evolutionary advantage for macrophages as cellular reservoirs.

## Macrophage and HIV-1 Interplay With Host Immune Responses

### Infected Macrophages Resist HIV-1 Cytotoxic Effects

**FIGURE 2:**
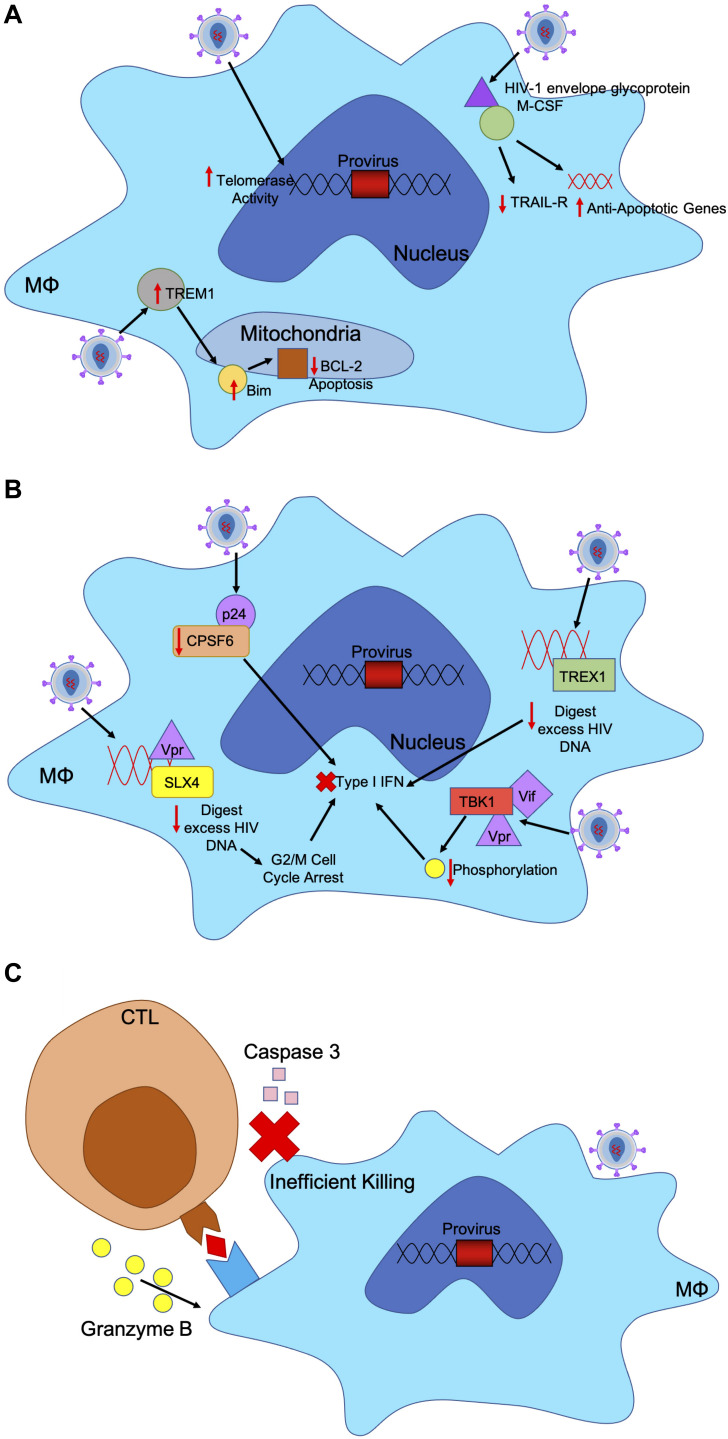
HIV-1 has evolved mechanism to evade host immune responses in macrophages. **(A)** HIV-1 infected macrophages are refractory to the cytopathic effects of viral infection. Infected cells usually undergo apoptosis or cell-mediated killing upon viral infection. HIV-1 upregulates certain factors to evade apoptosis. HIV-1 envelope glycoprotein binds to M-CSF to downregulate TRAIL-receptor and upregulate anti-apoptotic genes. HIV-infected macrophages express higher levels of TREM1, which upregulates and translocates Bim to the mitochondria to downregulate BCL-2 induced apoptosis. Additionally, HIV-infected macrophages have increased telomerase activity, triggering less DNA damage and sustained infection. **(B)** HIV-1 induces mechanisms to evade IFN responses in infected macrophages through accessory proteins. TREX1 binds to excess HIV DNA during reverse transcription to suppress IFN responses. HIV-1 capsid binds to and removes CPSF6 and cyclophilins to silence downstream innate sensors of IFN pathways. HIV-1 Vif and Vpr bind to TBK1, preventing phosphorylation and downstream IFN induction. HIV-1 Vpr also binds to SLX4 complex with reverse transcribed HIV-1 DNA, like TREX1 to degrade nucleic acids and increase cellular replication stress, leading to G2/M cell cycle arrest and prevent IFN response. **(C)** CTLs recognize peptide antigens presented by MHC-I and release cytotoxic particles to kill infected cells. HIV-1 infected macrophages are resistant to CTL killing as CTLs require both granzyme B and caspase-3 to efficiently kill HIV-1 infected macrophages unlike CD4+ T cells which only need granzyme B. CTL binding to MHC-I only triggers release of granzyme B, which renders a much slower and less efficient killing of macrophages.

Upon HIV-1 infection, macrophages resist the cytotoxic effects of viral infection. Typically, infected cells undergo apoptosis or cell-mediated killing upon viral infection; however, HIV-1 has evolved mechanisms within infected macrophages to maintain cell health and sustain viral production (Figure 2A) (Coiras et al., 2009). In infected macrophages, HIV-1 envelope glycoprotein stimulates macrophage colony-stimulating factor (M-CSF) to downregulate tumor necrosis factor–related apoptosis-inducing ligand (TRAIL) receptor and upregulate anti-apoptotic genes allowing infected macrophages to remain unaffected (Swingler et al., 2007). In acute HIV-1 infection, a small number of infected microglia and macrophages expressed higher levels of Bim, which down regulates pro-apoptotic negative regulator Bcl-2, in the mitochondria both *in vitro* and *in vivo* (Castellano et al., 2017). These mechanisms used to downregulate key players in apoptotic mechanisms allow infected macrophages to persist.

Triggering receptor expressed on myeloid cells 1 (TREM1) has been shown to help mediate the resistance of macrophages to HIV-1 cytotoxicity. HIV-infected macrophages increase expression levels of Bcl-2, TREM1, BCLXL, MFN1 and MFN2, translocating Bim to the mitochondria and decreasing Bcl-2 induced apoptosis. TREM1 silencing leads to significant increase in expressions of BAD and BAX, which results in apoptosis mediated by mitochondrial membrane disruption. TREM1 assist in upregulating BCL2 and mitofusins to avoid apoptosis (Campbell et al., 2019).

In addition, infected macrophages are capable of mediating apoptosis of CD4+ T cells through direct cell-to-cell contact, inducing ligands like FasL and tumor necrosis factor to selectively deplete CD4+ T cells (Badley et al., 1997). This resistance to the cytotoxic effects of HIV-1 enables macrophages to be reservoirs that can produce virus long term. Other mechanisms have been elucidated that may protect infected macrophages from the cytotoxic effects of HIV-1 infection, which suggests protection of macrophages as reservoirs by HIV-1. HIV-1 infected MDMs have been shown to have protection against oxidative stress through increased telomerase activity in comparison to uninfected MDMs. The infected MDMs had significantly less DNA damage when HIV-1 induced telomerase activity, which suggests the evolutionary advantages of sustaining macrophage infection (Reynoso et al., 2012). While mechanisms as to why infected macrophages are resistant to many HIV-1 cytotoxic effects are still under question, this highlights a potential strategy by HIV-1 in protecting macrophages and allowing them to be better suited, immunologically safe sanctuaries for sustained infection.

### HIV-1 Induces IFN Resistance in Macrophages

HIV-1 viral fitness can be determined by resistance to type 1 interferon. Transmitted viruses with higher IFNα2 and IFNβ resistance are found to more likely replicate than those not resistant to interferon (Iyer et al., 2017). These viruses are capable of replicating and spreading more efficiently in CD4+ T cells when faced with the innate immune response. Interferon-induced transmembrane proteins (IFITMs) retract viral entry into macrophages and CD4+ T cells, which can be evaded through co-receptor binding by HIV-1 glycoproteins. Transmitted founder viruses are uniquely resistant to the antiviral activities of IFITMs, which suggests the higher fitness of these HIV-1 virions (Foster et al., 2016).

In primary macrophages, HIV-1 has been able to replicate without signaling the innate immune system even though reverse transcription uses genomic RNA that may trigger pattern recognition receptors (PRR) (Figure 2B) (Rasaiyaah et al., 2013). Upon entry into macrophages, HIV-1 capsid depletes cofactor cleavage and polyadenylation specificity factor subunit 6 (CPSF6) and cyclophilins to silence innate sensors triggering type 1 IFN response. This allows HIV-1 to remain hidden in primary macrophages (Rasaiyaah et al., 2013). In both macrophages and CD4+ T cells TREX1 was found to also suppress HIV-1 triggered IFN responses by binding to and digesting excess HIV DNA, as well as blocking IFN signaling cascade through STING, TBK1, and IRF3 (Yan et al., 2010).

Moreover, HIV-1 accessory proteins Vpr and Vif are responsible for innate immune evasion in macrophages and dendritic cells. While HIV-1 viral RNA is detected by PRRs, Vif, and Vpr bind to TANK-binding kinase 1 (TBK1) and prevent phosphorylation, thereby blocking IFN induction (Harman et al., 2015). In addition, HIV-2 accessory protein Vpx eliminates the antiviral responses by degrading SAMHD1 in monocytes, macrophages and dendritic cells. SAMHD1 is responsible for restricting dNTPs available in the cytosol, thereby limiting the ability of HIV-1 to replicate in myeloid cells (Plitnik et al., 2018). SAMHD1 has been associated with negatively regulating IFN responses through unknown mechanisms (Rice et al., 2009). Lower levels of dNTPs and reverse transcription in myeloid cells may assist in preventing detection by IFN triggering responses. Consequently, HIV-1 does not have Vpx but Vpr to assist in downregulating antiviral responses. Vpr induces cell cycle arrest at the G2 to M phase transition through various mechanisms, therefore allowing HIV-1 to avoid detect by IFN responses (Andersen and Planelles, 2005; Bregnard et al., 2014; Laguette et al., 2014). Vpr binds to structure-specific endonuclease SLX4 complex to avoid innate immune surveillance. SLX4 complex is necessary for nucleic acid metabolism and repair in DNA lesions. When bound to by Vpr, SLX4 signals downstream cellular replication stress, which leads to G2/M cell cycle arrest (Laguette et al., 2014). SLX4 complex binds directly to newly reverse transcribed HIV-1 DNA and Vpr to inhibit HIV-1 driven type 1 IFN. SLX4 degrades excess nucleic acids derived from HIV-1 reverse transcription that may trigger PRRs and downstream IFN (Bregnard et al., 2014).

While HIV-1 inhibits type I IFN, HIV-1 stimulates interferon-stimulating genes (ISG) in macrophages by reverse transcription of HIV-1 RNA, triggering the RIG-I. HIV-1 Tat stimulates RIG-I, MAVS, IRF1, and IRF7, causing ISG IFN-induced protein with tetratricopeptide repeats (IFIT) to inhibit HIV-1 replication and production. By limiting HIV-1 replication, HIV-1 is rendered quiescent in macrophages, causing replication to persist at low levels and for infected macrophages to evade further immune responses (Nasr et al., 2017). Still, some HIV-1 viruses are highly susceptible to IFN responses, therefore HIV-1 has evolved mechanisms to evade the innate immune and antiviral state during infection. In macrophages specifically, HIV-1 has evolved mechanisms that avoid triggering antiviral responses and provide an immunologic sanctuary to protect HIV-1 against immune responses and sustain infection.

### CTL Resistance in Macrophage Reservoirs

HIV-1 infection involves successful evasion of multiple antiviral mechanisms levied by both the innate and adaptive immune systems. While HIV-1 encodes accessory proteins that counteract cellular restriction factors of the innate immune system, HIV-1 must also escape detection from responses triggered by the adaptive immune response. As an intracellular pathogen, HIV-1 must evade attacks from antibodies and cytotoxic cells, such as CD8+ Cytotoxic T lymphocytes (CTLs) (Arcia et al., 2017). CTLs recognized cells expressing peptide antigens through major histocompatibility complex class I (MHC-I) on all nucleated host cells, allowing these cytotoxic cells the ability to identify and attack infected cells. With such a potent adaptive immune response, HIV-1 established infections in CD4+ T cells and macrophages have evolved mechanisms to evade CTL responses (Clayton et al., 2018). HIV-1 specific-CTLs are able to detect HIV-1 viral peptides expressed by MHC-I expressed on infected CD4+ T cells, prompting CTLs to attacked the infected cells. Studies in non-human primates models show that upon antibody depletion of CD8+ T cells, there is a spike in viral replication and viral load found in peripheral blood (Rainho et al., 2015). The critical role of CTLs in controlling HIV-1 infection is seen in HIV-1 elite controllers that maintain very low viral loads without ART. These elite controls have CTLs capable of proliferating more efficiently and producing higher levels of Perforin and Granzyme B (Migueles et al., 2002; Walker-Sperling et al., 2014).

While CTLs are one of the main immune responses involved in controlling HIV-1 infection, high genetic diversity and high mutation rate of the HIV-1 genome assist in evasion of CTL surveillance. CTLs impose one of the strongest selective pressures on HIV-1, thereby being the predominant driving force for HIV-1 evolution (Price et al., 1997; Jones et al., 2004; Allen et al., 2005; Streeck et al., 2006; Liu et al., 2011). Mutations in the HIV-1 genome due to selective pressure of the CTL immune response have been termed CD8 escape mutants. Immunodominant epitopes derived from HIV-1 have been correlated with a strong CTL response, which suggests CTLs with gag-restricted epitopes have higher response activity (Cao et al., 2003). During acute HIV-1 infection, virus-specific CTLs emerge with the ability to target HIV-1 and to secrete inflammatory molecules against the virus. In acute infection, CTLs deplete HIV-1 infected CD4+ T cells, allowing CTLs to partially control the infection (Allen et al., 2005).

Continued CTL responses allow for HIV-1 evolution by selecting for viral escape mutants capable of avoiding the adaptive immune response against HIV-1. The escape mutations are located in critical HIV-1 genome sites presented by MHC-1 as peptides for recognition by CTLs and restricted to those used by MHC-I. In chronic HIV-1 infection, CTL killing targets distinct epitopes in the *gag* region of the HIV-1 genome and structural proteins in *nef*. Over time, mutations in these *gag* epitopes are rapidly selected, allowing the virus to escape CTL immune responses. Cao et al. (2003) have found patients partaking in ongoing ART are protected against the evolution of viral escape mutants due to limited viral replication. For those not on ART, when the viral load decreases due to immune responses, viral strains with CD8 escape mutants comprise the majority of the viral population.

In addition, HIV-1 accessory protein Nef has been shown to assist HIV-1 in successful evasion of CTLs through mislocalization and degradation of MHC-I in the infected cell (Collins et al., 1998). Nef directly acts as an anchor to stabilize the bond between MHC-1 and the clathrin-dependent trafficking through clathrin adaptor protein-1 (AP-1) (Roeth et al., 2004). AP-1 binding of MHC-I directs MHC-I to degradation more rapidly, thereby reducing the amount of MHC-I found on the cell surface (Collins and Collins, 2014). By downregulating MHC-I on CD4+ T cells and macrophages, CTLs are not presented with HIV-1 antigen peptides, which allows HIV-1 infected cells to escape immune surveillance.

Most of the attention on the impact of CTL activity has been on HIV-1 infection of CD4+ T cells while the impact of CTL on HIV-1 infection of macrophages has been understudied. Clayton et al. (2018) found that HIV-infected macrophages are inefficiently killed by CTLs due to an intrinsic resistance of macrophages to CTL killing (Figure 2C). CTLs require both granzyme B and caspase-3 to efficiently kill HIV-1 infected macrophages, while granzyme B is not needed in CTL-mediated killing of CD4+ T cells, allowing HIV-1 more resistance to CTLs when hidden in macrophages. For CTLs to effectively kill infected macrophages, longer cell-to-cell contact time and greater interferon responses are needed to trigger macrophage death (Clayton et al., 2018).

Rainho et al. (2015) demonstrated that SIV-specific CTLs are not able to effectively kill SIV-infected macrophages in macaques while CTLs are able to eliminate SIV-infected CD4+ T cells. While Nef is necessary to avoid CTL-mediated killing of CD4+ T cells, macrophages infected with *nef*-deficient HIV-1 variants were still refractory to CTL responses. This suggests macrophages have intrinsic characteristics and other mechanisms that provide resistance to CTLs (Collins and Collins, 2014; Rainho et al., 2015). Through a combination of viral escape mutations, accessory protein inhibition of cellular restriction factors, and capase-3-dependent killing, HIV-1 has been able to overcome the host’s adaptive immune responses. These determinants underscore the ability of HIV-1 to persist in the body. While HIV-1 itself has evolved mechanisms to evade CTL-killing, macrophages provide an immune safe haven for HIV-1 without the evolutionary need to evade CTL as macrophages are intrinsically refractory to affects from CTLs.

## Macrophage Reservoir Models, Studies and Eradication Strategies

### Humanized Mice: Mice Lacking T Cells Have Sustained HIV-1 Replication via Macrophages

To adequately study HIV-1 infection of macrophages *in vivo*, macrophages must be isolated and donated from elective surgeries and post-mortem tissue; however, such analysis does not provide a window for current infection or thorough exploration to outlying factors the host may contribute. Due to challenges in receiving human donor tissue to further study ART and viral reservoirs *in vivo*, humanized mice models have been designed to simulate HIV-1 infection in humans and study HIV-1 *in vivo* (Hermankova et al., 2003).

In creating these humanized mice models, researchers have trialed various strategies to design accurate models of HIV-1 infection *in vivo*. These strategies have determined suitable immunodeficient mice strains able to maintain HIV-1 infection; which human stem cells or tissue are engrafted; how mice immune cells are irradiated; and how human cells or tissue will be injected or engrafted into the mice. The engraftment of immunodeficient mice with live human cells and tissues has allowed for *in vivo* HIV-1 studies and HIV-1 preclinical research (Weichseldorfer et al., 2020). Scientists have used humanized severe combined immunodeficient (SCID) mice models to analyze HIV-1 infection and replication, as well as the efficacy of ART. These humanized SCID mice are repopulated with human hematopoietic stem cells (HSCs), human peripheral blood lymphocytes (PBLs), or with human tissue (McCune et al., 1988; Mosier et al., 1988; Namikawa et al., 1988). Tissue engraftment is typically from human fetal thymus or liver tissue (McCune et al., 1988). These mice models are capable of sustaining HIV-1 infection, replicating acute HIV-1 infection with limited CD4+ T cell lifespan (Weichseldorfer et al., 2020).

NOD-SCID (NSG) mice have gene mutations to minimize murine cells and better replicate the human immune system (Chen et al., 2009; Rongvaux et al., 2014). These mice models were eventually engrafted with human CD34+ HSCs to fully study HIV-1 persistence and latent reservoirs as the mice supported longer human cell lifespans and hematopoiesis (Brehm et al., 2010; Arainga et al., 2016). Additionally, these HSC engrafted humanized NSG mice developed their own lymphoid system from the HSCs, with lymphoid tissue capable of sustaining latent reservoirs upon HIV-1 infection. This model opened an avenue for in depth reservoir and latency research *in vivo.* Moreover, HIV latency can be duplicated in HIV-infected Human Immune System (HIS) mice under ART, opening a window into assessing HIV latency and distinct treatments that can eradicate HIV reservoirs (Churchill et al., 2016; Olesen et al., 2016; Deruaz and Tager, 2017). To assess these models, novel *in vivo* murine viral outgrowth assays have been developed to detect virus from undetectable viral load or to circumscribe whether elimination approaches are efficient in clinical trials (Metcalf Pate et al., 2015; Charlins et al., 2017; Descours et al., 2017).

Humanized mice models have allowed for the study of myeloid reservoirs in combination with T cells or as a single reservoir in Bone marrow, lymphoid, thyroid (BLT) mice. These mice are transplanted with hematopoietic stem cells and are responsive to ART (Arainga et al., 2017). BLT mice infected with HIV-1 have detectable HIV-1 DNA and RNA from T cells and macrophages (Honeycutt et al., 2016). Recent studies utilizing T-cell-only (TOM) and myeloid-only (MOM) HIS mice have revealed latent CD4+ T cell and myeloid HIV reservoirs can develop independently (Honeycutt et al., 2013; Honeycutt et al., 2017). In a specific mice model, NOD/SCID mice were engrafted with only hematopoietic stem cells but unable to support lymphocytes, which allowed for only myeloid cell growth. Upon infection with macrophage tropic HIV-1, efficient infection and sustained replication of HIV-1 persisted in these Myeloid-Only Mice even in the presence of ART (Honeycutt et al., 2016). Furthermore, HIV disseminated extensively to various tissue compartments in these mice—including the brain—heavily suggesting that myeloid cells can remain a source of HIV composition *in vivo* (Baxter et al., 2014; Calantone et al., 2014; Honeycutt et al., 2017). Unfortunately, these MOM models have a short turnover of around 1 day for human macrophages, greatly underestimating the half-life of macrophages found in the human body. This emphasizes that conditions even in animal models do not fully characterize *in vivo* infection of HIV-1 in humans.

Although there are some limitations shown in murine models, they address the existence of macrophage reservoirs in sustaining persistent HIV-1 infection that is not available from human tissue studies. Further advancement of murine qVOA studies may elucidate detection of HIV-1 in low levels from quiescent macrophage reservoirs in future studies. These murine models are an invaluable resource in the limitation of human *in vivo* studies and allow for further investigation into macrophage reservoirs and their contribution to HIV-1 pathogenesis.

### Tissue Macrophages Roles as HIV-1 Reservoirs

Tissue resident macrophages have been implicated in sustaining HIV-1 infection *in vivo* and producing virions *in vitro*, with the earliest evidence of HIV infection of macrophages in 1987 (Gartner et al., 1986; Perelson et al., 1997). The first confirmation highlighting the role of macrophages in HIV-1 persistence was described by Igarashi et al. (2001). When CD4+ T cells were depleted from macaques infected with SHIV, the infection was independently sustained by macrophages. After administration of a potent reverse transcriptase inhibitor, viral production was blocked in circulating CD4+ T cells but not in tissue macrophages, demonstrating that tissue macrophages can sustain HIV-1 infection alone (Igarashi et al., 2001).

Under cART, very low levels of free virus are found in plasma. ART-naïve patients were placed on cART treatment and monitored for HIV-1 RNA concentration in plasma. After 8 weeks of treatment, plasma viremia dropped below detectable levels and no infectious virus was found in PBMCs, yet there was still a secondary source of viremia. The residual viremia from these cART-suppressed patients has shown HIV-1 originates from more than the resting CD4+ T cell population, postulated to be tissue macrophages (Perelson et al., 1997). In a study by Bailey et al. (2006), through intensive sampling plasma and PBMCs from chronically infected patients, a predominant plasma sequence was found that was not related to those proviral sequences found in resting CD4+ T cells. The origin of the small number of clones, while not identified, has evidence of coming from a reservoir different than circulating CD4+ T cells (Bailey et al., 2006). Recrudescing viremia gives an opportunity to study the origin of rebounding viruses as they emerge from their cellular reservoir (Vibholm et al., 2019). While lymph nodes carry the majority of latent virus found in circulation, those that rebound during treatment interruption are not the same (Vibholm et al., 2019). Residual viremia from cART-suppressed patients is found to have genetically distinct genomes from proviruses found in resting CD4+ T cell, coming from an unidentified source (Brennan et al., 2009). From heavy sampling of residual viremia, some genomes originated from monocytes and unfractionated PBMC.

In a recent study by Andrade et al. (2020), rebound viremia was interrogated from chronically infected patients undergoing analytical treatment interruption (ATI). Single viral genomes were isolated and cloned. While most clones showed T-cell tropism, a small population of highly macrophage tropic clones were identified containing macrophage-specific markers from four patients. The group also enriched those M-tropic viruses from post-ATI plasma with macrophage specific markers CD14 antibody to confirm that M-tropic viruses had a macrophage origin as the viral envelopes contained CD14 from macrophage cell membrane. These results suggest macrophages are a viral reservoir that generate rebound viremia (Andrade et al., 2020).

While it remains unknown whether the source of viral rebound in patients treated during acute infection differs from chronic infection, there is evidence that HIV-1 establishes latent infection early on in both CD4+ T cells and macrophages. For Andrade et al. (2020) using molecular clock analysis, some of macrophage-tropic HIV-1 isolates were found to predate the start of cART and suggest that macrophage reservoirs are established in acute infection and assist in HIV-1 persistence. Furthermore, in many tissues like the lymph nodes, active reservoirs have been found as in quiescent CD4+ T cells and macrophages that have low levels of replication that sustain viral infection even in individuals that have progressed to AIDS (Embretson et al., 1993). For chronic phase infection, the diversity of HIV-1 viruses isolated during viral rebound is incredibly high, with plasma and proviral sequences intermingling and differing in phylogenetic trees (Bailey et al., 2006). This correlates with the differences in sizes of HIV-1 reservoirs as those treated during acute infection have much smaller reservoirs, while those treated during chronic infection have much larger HIV-1 reservoirs and therefore more diversity (Li et al., 2016).

Other studies use distinct strategies to find DNA and RNA of HIV-1 in several tissue compartments of animal models (Orenstein et al., 1997; Igarashi et al., 2001; Swingler et al., 2007; Cribbs et al., 2015). For instance, ART-suppressed humanized mice were infected with macrophage tropic virus to investigate the ability of virally infected cells to build reservoirs. The group detected HIV-1 DNA and RNA in mature macrophages in all treated mice (Arainga et al., 2017). Another study using a SIV/macaque model for HIV-1 encephalitis and AIDS demonstrated infected microglia persisted in the brain in presence of ART (Avalos et al., 2017). In addition, Avalos et al. (2017) exhibited that most suppressed macaques contained latently infected microglial cells and that virus produced by macrophage qVOA was infectious and replication-competent, which suggests microglia are capable of maintaining and reestablishing productive infection upon treatment interruption in macaques.

In human tissue, HIV DNA and RNA was found in the resident macrophages of the lungs, gut and male genital tract from ART-suppressed HIV-1 infected individuals. Cribbs et al. (2015) evaluated the presence of proviral DNA in alveolar macrophages from HIV-1 infected individuals under ART, displaying that alveolar macrophages harbor HIV-1 and may be a potential reservoir. Furthermore, a study has found evidence that human urethral tissue macrophage could constitute a principal HIV-1 reservoir. Ganor et al. (2019) demonstrated urethral penile tissue macrophages have integrated HIV-1 DNA, RNA, proteins, and intact virion. These macrophages were stimulated with lipopolysaccharide on urethral single cells suspension from HIV-1/cART individuals and showed reactivation of HIV-1 through modified qVOA supporting that tissue macrophages have replication-competent virus from integrated HIV-1 DNA, which was not evident in CD4+ T cells stimulated with PHC (Ganor et al., 2019). Thus, macrophages are shown to be the principal reservoir in urethral tissue containing integrated HIV-1 DNA that can induce outgrowth of replication-competent infectious HIV-1. Collectively, these findings have demonstrated that macrophages sustain latent HIV-1 infection and assist in HIV-1 persistence. With this, macrophages are shown to be a viable reservoir that contribute to obstacles in eradicating HIV-1.

### Latency Reverse Agents and Challenges of Studying Macrophages Reservoir

While ART is capable of limiting HIV-1 replication, ART alone does not eradicate HIV-1 as these inhibitors are not able to directly attack the latent HIV-1. Several latency reversing agents (LRAs) have been identified and used to reactivate latent HIV-1 from their proviral state in cellular reservoirs *in vitro* and *ex vivo* (Nakamura et al., 2013). Many of these LRAs are designed to reactivate HIV-1 in latent CD4+ T cells, so accessing and reactivating latent virus in macrophages is still largely unknown. Macrophage reservoirs are present in compartmentalized tissues, including the CNS, which may contribute to the lack of efficacy in eliminating infected cells and the failure of reactivating latent proviral DNA as these tissues remain largely unaffected by LRA compounds. The topic of HIV latency within macrophages is debatable, highlighting the need for LRAs on this cell type.

There are six primary groups of LRAs categorized by their mechanism of action within the host cell. These six groups can be categorized as histone post-translational modification modulators, non-histone chromatin modulators, NF-κB stimulators, TLR agonists, extracellular stimulators, and miscellaneous, which is comprised of unique and uncommon compounds (Abner and Jordan, 2019). The histone post-translational modification modulators group includes histones methyltransferase (HMT) and histones deacetylase inhibitors (HDACi). These compounds function by regulating histone tail modulation of nucleosomes with integrated HIV-1 and reversing the latent provirus to active. A few drugs previously approved for cancer treatment are currently being investigated as potential LRAs, such as valproic acid, vorinostat, panobinostat, and romidepsin. Deeks (2012) demonstrated a potential therapeutic approach called “Shock-and-Kill” that accesses latent virus in infected cells by forcing them to become active from a quiescent state, thereby killing active virus and infected cells to eliminate the viral reservoir (Nakamura et al., 2013). Archin et al. (2012) tested the shock-and-kill approach in a clinical trial using vorinostat that activated viral replication in HIV-1+ individuals (Lanktree et al., 2011; Archin et al., 2017). Activation of HIV-1 genes was validated by a noticeable upregulation of viral RNA synthesis. Subsequently, other clinical trials were conducted using vorinostat for longer periods of time, which confirmed an increase of HIV-1 cell-associated RNA in circulating resting CD4+ T cells and activated the latent CD4+ T cell reservoir (Archin et al., 2014a, b, 2017; Arcia et al., 2017).

Some studies administered HDACi *in vitro* in the monocytic U1 cell line and MDMs, which have shown not only reactivation but decreased HIV-1 release and degradation of viral particles (Rasmussen et al., 2013; Campbell et al., 2015); however, HDACis at higher doses can cause numerous side-effects and are considered weak LRAs. Another particularly important LRA category is NF-κB stimulators, which are Protein Kinase C (PKC) pathway agonists leading to upregulation of transcription factor NF-κB to reactivate HIV-1. Several PKC agonists, like prostratin and ingenols, have also been shown to effectively reactivate latent HIV-1 targeting different pathways in T cells, as well as in monocytic cell lines (Fernandez et al., 2013; Abreu et al., 2014; Darcis et al., 2015; Jiang et al., 2015).

Due to the deficiencies of administration of only one LRA, combinatory “shocks” have taken place in other studies (Margolis and Hazuda, 2013). Combining HDAC inhibitors themselves, or with other classes of LRA drugs, shows increased efficacy at latency reversal since it generates a highly synergic action to reactivate HIV-1. Darcis et al. (2015) combined Bromodomain and Extraterminal (BET) bromodomain inhibitors (BETi) with NF-kB inducing agents, and *ex vivo* models have shown their combination led to synergistic activation of HIV-1 expression at the viral mRNA and protein levels. The combination used *in vitro* of the PKC agonists and P-TEFb-releasing agents administrated together on HIV-1 post-integration latency model cell lines of T-lymphoid and myeloid lineages also have shown the synergistic reactivation of viral components. These results constitute one of the first demonstrations of combined LRA therapies and has shown promising strategies in reducing the size of the total HIV-1 reservoir (Darcis et al., 2015).

Most studies with LRAs evaluate the efficacy of reverse latency in blood compartments, focusing on their effects on resting memory CD4+ T cells with latent provirus (Spivak et al., 2016; Gupta and Dixit, 2018; Spivak and Planelles, 2018). In contrast, the subject regarding macrophages in the CNS is just beginning to be thoroughly explored. The reservoir studies of the CNS are considered particularly challenging due to several characteristics of the environment. First, the CNS is protected by the blood brain barrier and the Choroid plexus, which limits drug access. The CNS is an immune privileged environment, complicating assessment of drug penetration and effectiveness. In considering if an LRA could possibly clear these viruses, there may be an issue with inflammation and neurotoxicity as negative side effects in the brain compartment (Marban et al., 2016). Additionally, the difference in macrophage cell markers and receptors impact drug efficiency. Despite these impediments, there has been evidence that administration of LRA would have beneficial eradication effects on macrophage reservoir.

Campbell et al. (2015) have shown some differences between the effects of HDACis in CD4+ T cells and macrophages, as well as microglia. For macrophages, HDACis could induce autophagy pathways *in vitro* and achieves the inhibition of HIV-1 replication without reaching cell death. In contrast in CD4+ T cells, the administration of HDACis have the ability to reactivate the quiescent transcriptional viruses to be killed. Gama et al. (2017) described a possible effective administration of LRAs on virally suppressed macaques infected with SIV where reactivation of the virus in CNS was observed. The study showed an increase of viral load within the Cerebral Spinal Fluid, which highlights the brain as an important viral reservoir compartment (Gama et al., 2017).

Gray et al. (2016a; 2016b) have studied the toxicity of several commonly used LRA, such as Panobinostat, Romidepsin, and vorinostat, in primary astrocytes and MDMs. The results demonstrated that the administration of therapeutic concentrations was not toxic for these cells. Also, the group noted a greater viral reactivation in primary astrocytes, which could suggest a possibility of activation of latently infected cells in the CNS (Gray et al., 2016a, b). Jiang et al. (2015) utilized combinations of LRAs on well-studied HIV-1 latency lymphocyte and promonocyte cell culture models. The results of the study showed a potential synergism that englobe PEP005 on reactivate latency on J-Lat A1 cells and U1 cells (Jiang et al., 2015). The use of LRAs on macrophages is still in its early stages of research, but initial data and synergism reveal a promising approach that may to advance HIV-1 eradication strategies.

The HIV-1 reservoir field faces several challenges to elucidate mechanisms of HIV-1 persistence. The establishment of cellular reservoir can occur in the early days after individuals are infected. Considering that ART can start as early as a few months after the day of infection, HIV-1 integrates its genome into the host genome early in infection. Host cells will carry the genetic information necessary to produce new infectious virions for the cell’s lifetime, and the virus will persist at the individual system. For that reason, reservoir studies have been focused on the development tools to target and measure the cellular reservoir, and specifically latent resting memory CD4+ T cells. The lack of specific markers that could distinguish latently infected from uninfected cells represents a significant impediment in the design of a cure. The study of macrophage reservoirs is even more challenging. Unlike circulating CD4+ T cells, macrophages reside in every tissue in the human body and consequently become a unique population phenotypically distinct from others (Jambo et al., 2014). In addition, the complexity of these reservoir is due to their resistance of cytopathic effect, which leads to a long-lived macrophage and contribute to one of the barriers for elimination. Identification and use of LRAs for eradication of macrophage reservoirs has been limited through sanctuary tissue states. While certain LRAs for macrophages, like lipopolysaccharide, have been identified, their efficiencies are not well understood and limit the advancement of cure strategies in targeting cell specific reservoirs (Ganor et al., 2019).

## Conclusion

Since the discovery of HIV-1, a cure for HIV/AIDs has eluded researchers. With the introduction of ART to HIV-infected individuals, HIV-1 has become a chronic disease in which focus has shifted from treatment to finding a sterilizing or functional cure. With 38 million individuals currently living with HIV-1, efforts to fully understand cellular reservoirs and mechanisms of viral persistence are vital to reaching this goal. Attention will need to shift to further investigate myeloid reservoirs as literature over the past few decades has shown macrophages are not only susceptible to HIV-1 infection but also assist in sustaining viral persistence, even when CD4+ T cells are not present. A comprehensive understanding as to how HIV-1-infected macrophages are capable of avoiding both innate and adaptive immune responses, and the cytotoxic effects of viral infection, is necessary to discover ways to target and eradicate macrophage reservoirs. While many discoveries as to the mechanisms for why macrophages have been made, much of how macrophage reservoirs contribute to HIV-1 persistence remains unknown. Macrophages heterogeneity and biological characteristics makes it difficult to study macrophages’ whole population due to differences in susceptibility and infection based on tissue compartmentalization. Such characteristics affect the outcomes for viral persistence and efficient ways to eradicate macrophage reservoirs. While there has been accumulating evidence supporting macrophage reservoirs contribution to viral persistence, much of their mechanisms and contributions to viral persistence remain unknown. Future studies must focus on ways to eliminate both the latent CD4+ T cell reservoir and macrophage reservoir for there to be a potential cure for HIV-1. This, together with the stigma of HIV-1 infection, drives the rationale for developing a cure.

## Author Contributions

All authors listed have made a substantial, direct and intellectual contribution to the work, and approved it for publication.

## Conflict of Interest

The authors declare that the research was conducted in the absence of any commercial or financial relationships that could be construed as a potential conflict of interest.
